# Molecular Anatomy of Palate Development

**DOI:** 10.1371/journal.pone.0132662

**Published:** 2015-07-13

**Authors:** Andrew S. Potter, S. Steven Potter

**Affiliations:** Cincinnati Children’s Medical Center, Division of Developmental Biology, 3333 Burnet Ave., Cincinnati, OH, 45229, United States of America; The Roslin Institute, UNITED KINGDOM

## Abstract

The NIH FACEBASE consortium was established in part to create a central resource for craniofacial researchers. One purpose is to provide a molecular anatomy of craniofacial development. To this end we have used a combination of laser capture microdissection and RNA-Seq to define the gene expression programs driving development of the murine palate. We focused on the E14.5 palate, soon after medial fusion of the two palatal shelves. The palate was divided into multiple compartments, including both medial and lateral, as well as oral and nasal, for both the anterior and posterior domains. A total of 25 RNA-Seq datasets were generated. The results provide a comprehensive view of the region specific expression of all transcription factors, growth factors and receptors. Paracrine interactions can be inferred from flanking compartment growth factor/receptor expression patterns. The results are validated primarily through very high concordance with extensive previously published gene expression data for the developing palate. In addition selected immunostain validations were carried out. In conclusion, this report provides an RNA-Seq based atlas of gene expression patterns driving palate development at microanatomic resolution. This FACEBASE resource is designed to promote discovery by the craniofacial research community.

## Introduction

Failure of midline fusion resulting in cleft lip with/or without cleft palate is one of the most common birth defects [1–4]. The palate separates the oral and nasal cavities, with a bony anterior, and a muscular posterior that plays an important role in human speech and swallowing.

The mouse provides a useful model for the study of palate development. The palate develops primarily from neural crest [5], but with important paraxial mesoderm contribution to posterior muscle [6], and critical mesenchymal-epithelial interactions [7]. The mouse palatal outgrowths form initially at around E11.5 and extend vertically beside the tongue [7]. The palatal shelves then elevate to a horizontal position and at around E14.5 undergo fusion, leaving a medial edge epithelium (MEE) [8] surrounded by mesenchyme. This MEE then undergoes dissolution, leaving confluent mesenchyme in the forming palate. For a more detailed description of craniofacial development, including extensive color coded figures, refer to FACEBASE.ORG. Impressive progress has been made in defining the genetic regulatory network that drives palate development [7,9,10]. Much of our understanding derives from the study of mice with targeted mutations, finding key transcription factors, growth factors and receptors required for normal palatogenesis. Nevertheless, it is clear that our current understanding is far from complete.

Global expression studies, using microarrays or RNA-Seq, can provide an important discovery function, identifying previously overlooked pathways that might play important roles in craniofacial development. An early work used 6800 gene Mu11kB Affymetrix microarrays to examine gene expression in vertical, horizontal, and fused palatal shelves, providing a large scale genomics view of this developmental progression [11]. Microarrays have also been used to describe gene expression patterns in wild type and mutant palates, thereby aiding the discovery of key noncanonical pathways downstream of TGFβ signaling during craniofacial development [12,13]. In addition, microarrays have been used to examine early events in craniofacial development, including the creation of a comprehensive dataset of gene expression patterns of the mandibular, maxillary and frontonasal prominences of the mouse embryo from E10.5 to E12.5, using tissue isolated by manual dissection [14].

The FACEBASE consortium was established in part to provide a resource center for craniofacial researchers [15] (FACEBASE.ORG). One function of FACEBASE is to create a molecular anatomy of craniofacial development. To this end we have extended previous global studies of gene expression during craniofacial development by incorporating laser capture microdissection (LCM), which allows the high resolution analysis of specific compartments, compared to earlier work using manual dissection methods. In addition, the FACEBASE studies include RNA-Seq, with its many advantages over microarrays.

We previously reported a gene expression atlas of early craniofacial development, carrying out LCM/RNA-Seq gene expression profiling of the multiple progenitors of the face, as well as flanking tissues, from E8.5 to E10.5 [16]. In this report we extend this work to examine the forming palatal shelves at E14.5, shortly after medial fusion. The palatal shelves were divided into medial and lateral, as well as oral and nasal compartments, to provide a detailed view of region specific gene expression patterns. Further, both the anterior and posterior sectors of the palate were examined. The results help to identify novel compartment specific markers, assist in the design of useful compartment specific transgenic CRE and GFP mouse lines, aid in the discovery of novel flanking tissue interactions, and provide a global view of all transcription factors, growth factors and receptors expressed during this stage of palate development.

## Results and Discussion

**Fig 1:**
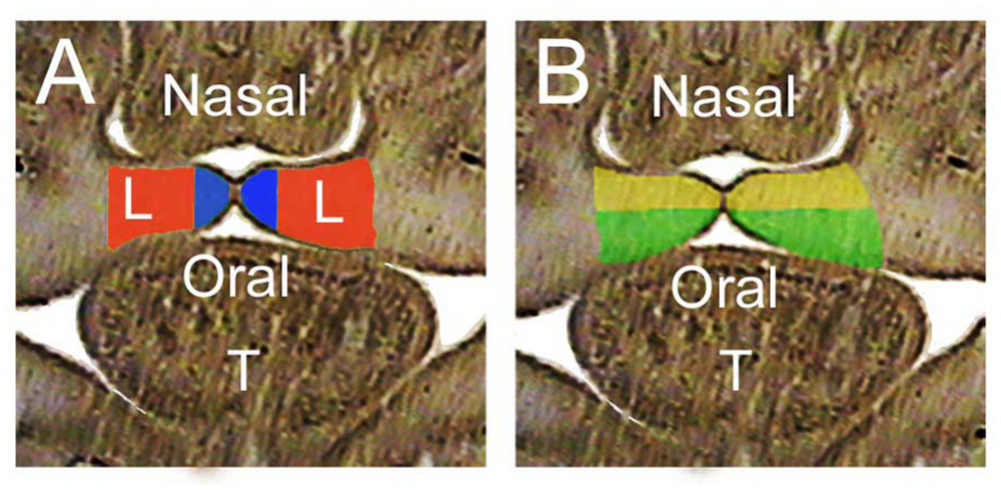
Diagram of compartments isolated by Laser Capture Microdissection. A. Medial (blue) and lateral (red) compartments of E14.5 palatal shelves. B. Oral (green) and nasal (yellow) compartments of palatal shelves. T marks tongue. Nasal region is above and the oral is below the forming palate.

To create a more complete molecular definition of the gene expression programs active in the E14.5 forming palate we used a combination of LCM and RNA-Seq. Both anterior and posterior regions of the palate were characterized, and each was partitioned into medial and lateral as well as oral and nasal compartments (Fig 1). Biological triplicates were examined for each sample type, excepting one quadruplicate, with a total of 25 RNA-Seq datasets produced.

### Anterior posterior

**Fig 2:**
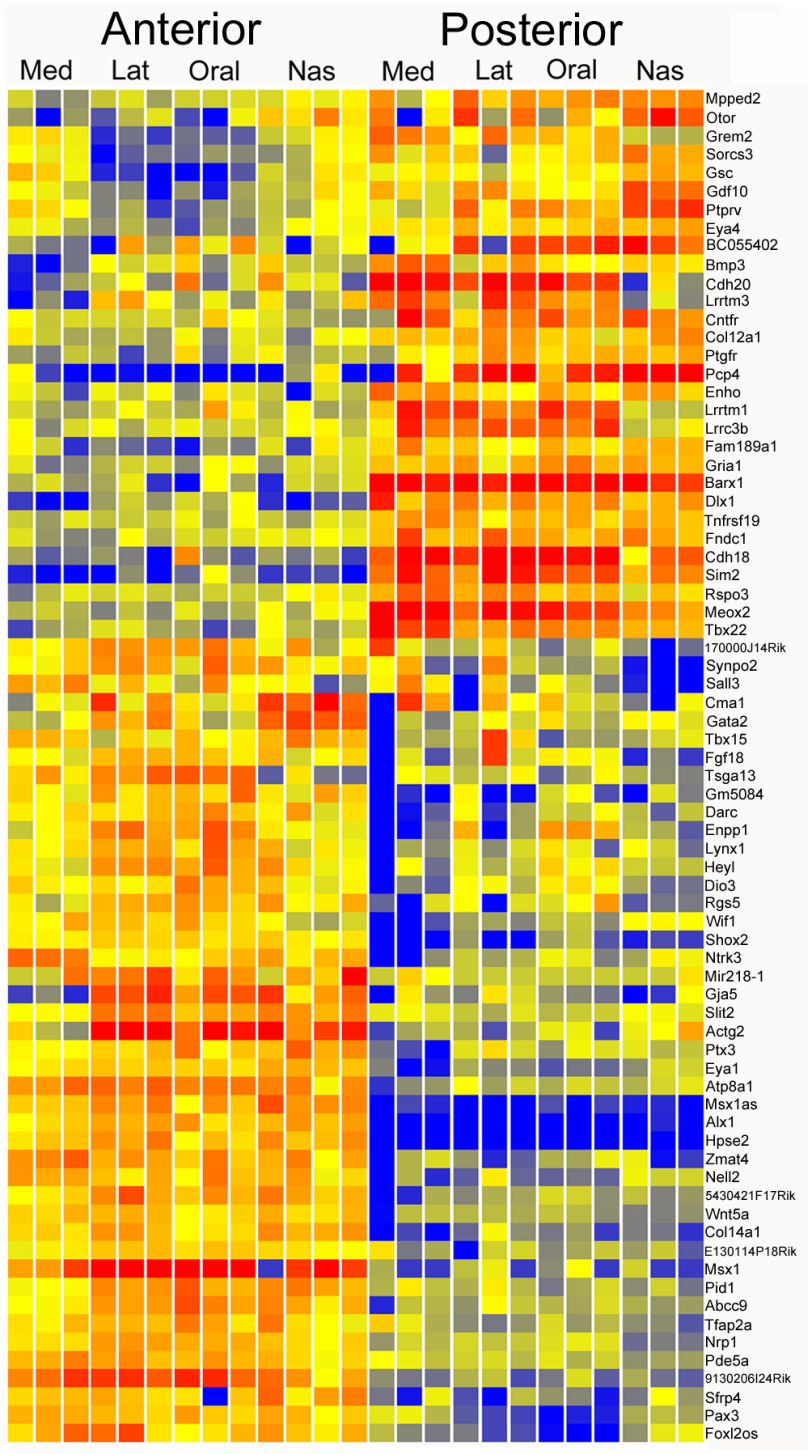
Comparison of palate gene expression patterns in the anterior and posterior compartments. Pooled comparison of all anterior versus all posterior compartments. This heatmap shows genes with greater than three fold change in comparing all combined anterior versus all combined posterior compartments.

We first searched for genes with differential expression along the AP axis during palate development. We used GeneSpring to carry out an Audic-Claverie pooled analysis comparison of the anterior and posterior compartments, requiring robust expression, with a minimum of 10 RPKM in six samples, P < 0.05 and Fold Change (FC) > 2. This analysis compares all combined anterior versus all combined posterior compartments, identifying 163 genes with differential expression (S1 Table). A more stringent screen, with FC > 3, gave 74 genes (Fig 2).

The resulting dataset showed strong agreement with previously published observations. For example, differentially expressed transcription factors included *Msx1* (19FC)(Zhang, 2002 #2)(Hilliard, 2005 #3), and *Shox2* (13FC)(Hilliard, 2005 #3)(Yu, 2005 #5) in the anterior domain, and *Barx1* (25FC)(Welsh, 2009 #1), *Meox2* (12FC)(Li, 2007 #6) and *Tbx22* (8FC)(Liu, 2008 #7) in the posterior domain. Additional transcription factor genes with differential (FC > 3) AP expression included *Pax3* (6FC), *Tbx15* (3FC), *Heyl* (3FC), *Gata2* (3FC), *Dlx1* (12FC), *Eya1* (5FC), *Eya4* (3FC), *Alx1* (59FC), *Sim2* (32FC), and *Sall3* (3FC). Growth factor genes with differential AP expression included *Pdgfc* (2FC), *Bmp3* (3FC), *Cxcl12* (2FC), *Clec11a* (3FC), *Fgf18* (3FC), *Inhba* (2FC), *Wnt5a* (4FC) and *Gdf10* (4FC). It is interesting to note that most of the gene expression differences with FC >3 along the AP axis are consistently differentially expressed across each of the sampled medial, lateral, oral and nasal compartments (Fig 2).

### Anterior palate, lateral versus medial

**Fig 3:**
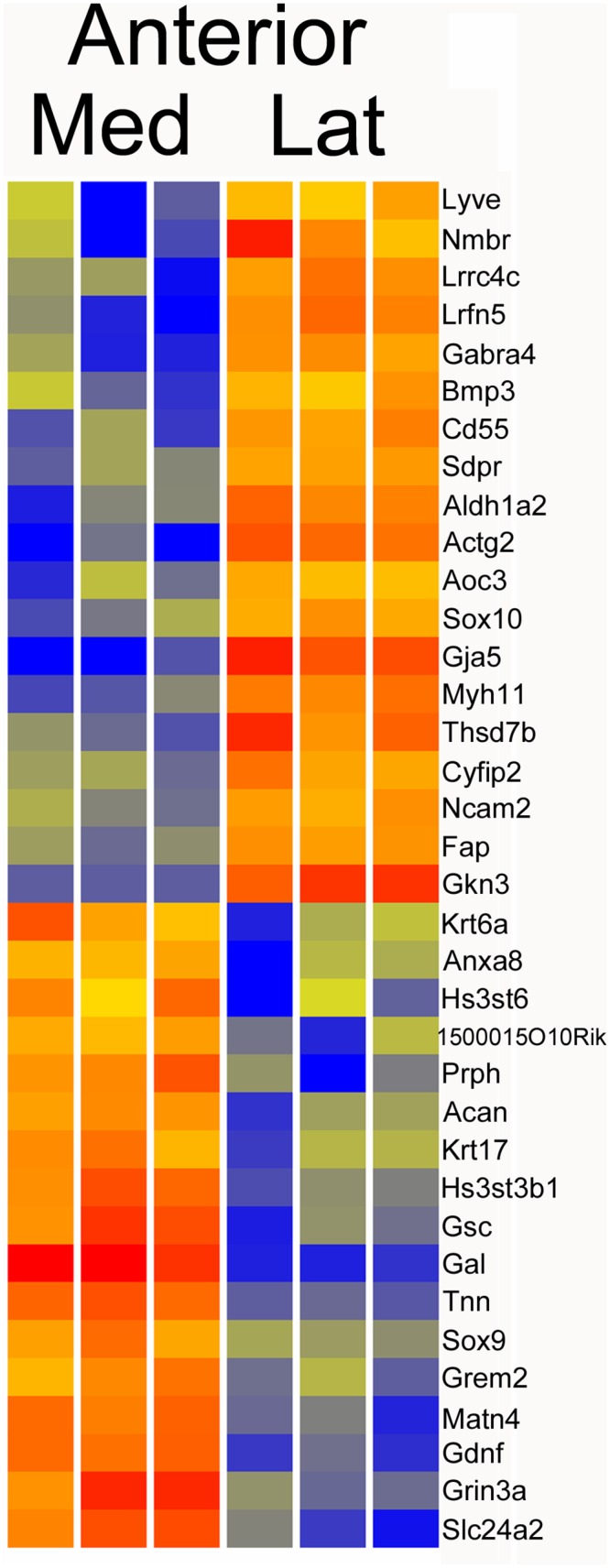
Heatmap of anterior medial versus anterior lateral compartments. Genes with greater than five fold change in comparing anterior lateral and medial compartments.

We compared the gene expression patterns of the anterior lateral and medial compartments, screening for expression over 5 RPKM in at least three samples, moderated t-test P < 0.05, and FC > 2, finding 232 genes with differential expression (S2 Table). A more stringent screen, requiring FC > 5, identified 36 genes, shown in the heatmap of Fig 3. Some of the strongest differences, considering both expression level and fold change, included *Gja5* (29FC), *Actg2* (23FC), *Nmbr* (14FC), *Aldh1a2* (10FC) and *Bmp3* (5FC) with higher expression in the lateral compartment, and *Gal* (54FC), *Gsc* (12FC), *Tnn* (12FC), *Prph* (11FC), *Grem2* (6FC), and *Sox9* (5FC) with higher expression in the medial compartment. Aldh1a2 synthesizes retinoic acid from retinaldehyde. *Nmbr* encodes the neuromedin B receptor. *Gsc* encodes a homeodomain transcription factor of known importance in craniofacial development. *Gal* encodes the galanin neuropeptide. *Prph* encodes a neuronal cytoskeletal protein. *Sox9* is a key driver of chondrogenesis. Bmp3, also known as osteogenin, induces bone formation, while *Grem2* encodes a BMP antagonist.

### Posterior lateral versus medial compartments

**Fig 4:**
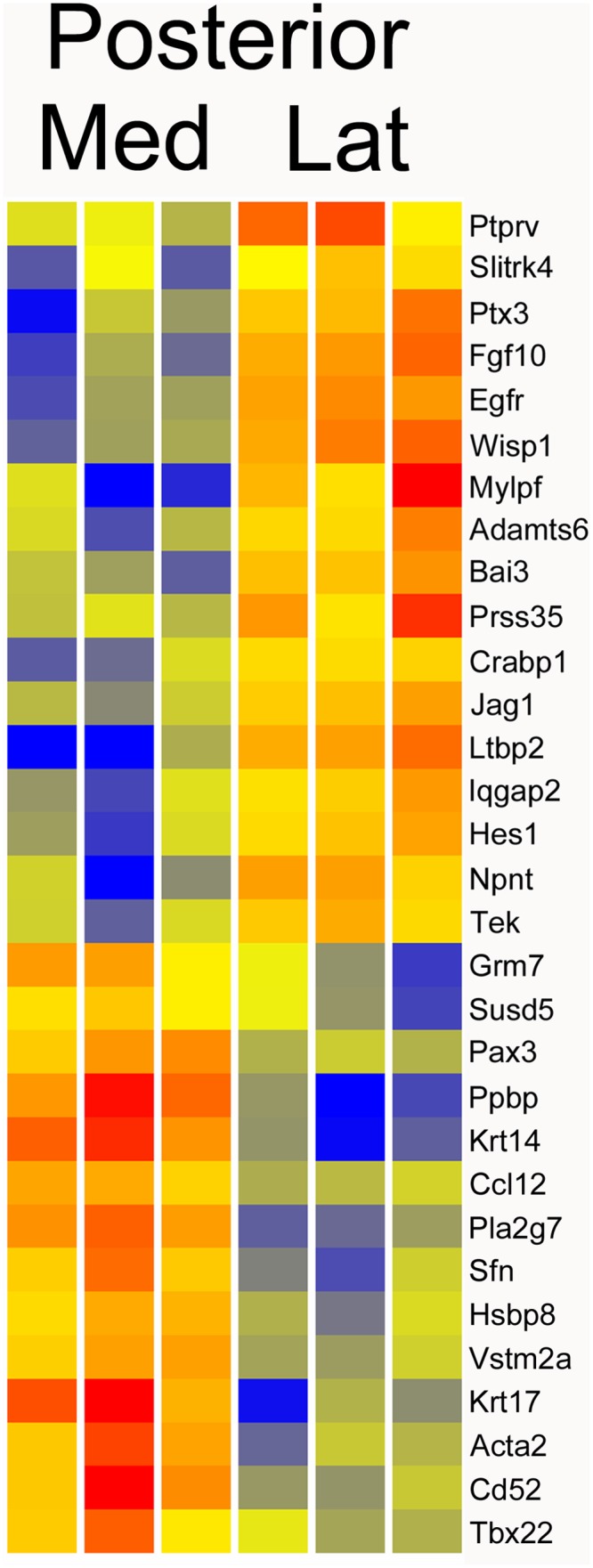
Heatmap of posterior medial versus posterior lateral compartments. Genes with greater than three fold change are shown. The list has a number of genes involved in growth factor signaling including *Fgf10*, *Egfr*, *Jag1*, *Npnt*, and *Grm7*.

A comparison of the posterior lateral and medial compartments, using the same parameters described for the anterior lateral versus medial, identified 79 genes with FC > 2 (S3 Table). A modest increase in stringency, to FC > 3, gave 31 genes (Fig 4). Some of the most interesting differences included *Ppbp* (18FC), *Pla2g7* (8FC), *Pax3* (4FC), *Fgf10* (8FC), *Ltbp2* (46FC), *Npnt* (7FC), *Egfr* (5FC), *Hes1* (4FC), *Jag1* (3FC). *Ltbp2* encodes a TGF-beta binding protein. Hes1 and Jag1 are involved in Notch signaling. *Ppbp* encodes a platelet derived growth factor and *Npnt* encodes nephronectin, an integrin binding protein that regulates expression of GDNF (glial derived neurotrophic factor). Of interest, several of the differentially expressed genes play important roles in growth factor signaling.

It is also interesting to note that there are relatively few genes in common between the anterior and posterior comparisons of medial and lateral compartments. Only ten genes are included in both lists of FC > 2, including *Npnt* (nephronectin), *Thbs2* (Thrombospondin2), *Egfr* (EGF receptor), *Wisp1* (Wnt1 inducible signaling pathway protein 1), and *Ptprv* (protein tyrosine phosphatase, receptor, type V).

### Oral versus nasal comparison in the anterior E14.5 palate

**Fig 5:**
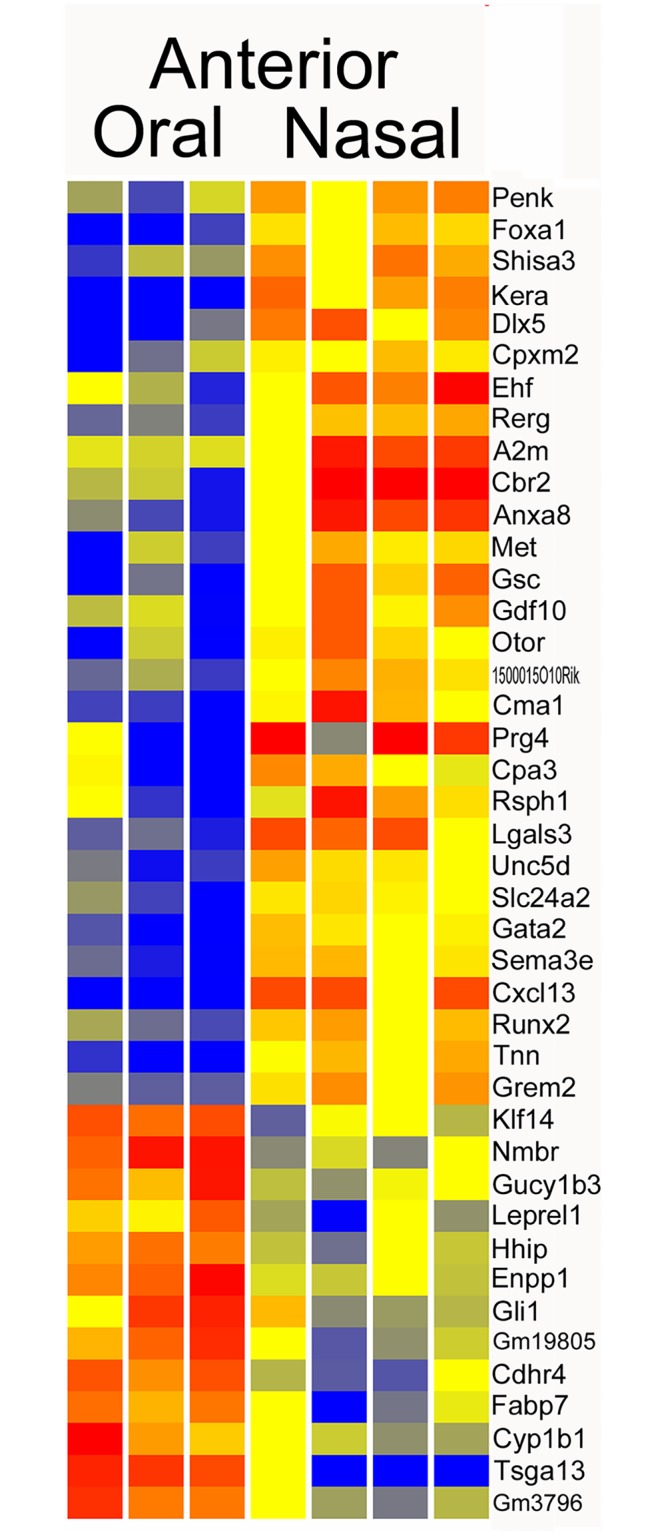
Heatmap of Anterior oral versus anterior nasal compartments. This list includes 42 genes with greater than three fold change.

A comparison of the oral and nasal compartments, in the forming anterior palate, gave 123 genes with differential expression (P < 0.05, FC > 2) (S4 Table). Increasing the stringency to require FC > 3 gave 42 genes (Fig 5). The genes with the most robust elevated expression in the nasal compartment, based on expression level and fold change, included *Cxcl13* (23FC), *Gsc* (13FC), *Dlx5* (11FC), *Cbr2* (8FC), *Tnn* (7FC), *Gata2* (6FC), *Cma1* (6FC), *Sema3e* (4FC), *Unc5d* (4FC), and *Runx2* (3FC). Genes with the most compelling elevated expression in the anterior oral compartment included *Tsga13* (11FC), *Nmbr* (5FC), *Klf14* (4FC), and *Enpp* (4FC).

### Posterior oral versus nasal compartments

**Fig 6:**
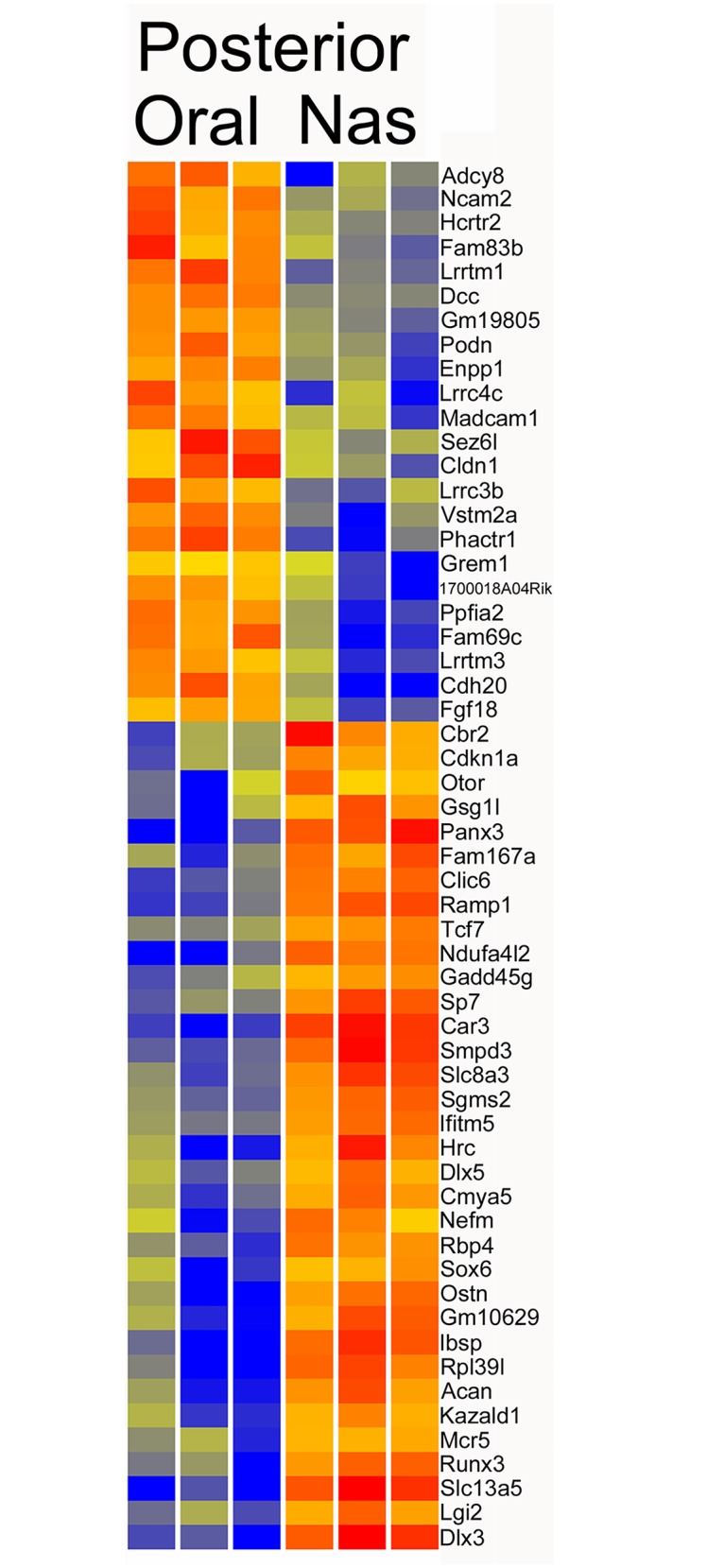
Heatmap of posterior oral versus posterior nasal compartments. Genes with greater than five fold change are shown.

The comparison of the posterior oral and nasal compartments found 311 differentially expressed genes (P < 0.05, FC > 2)(S5 Table). Increasing the fold change stringency to five gave 57 genes (Fig 6). Genes with elevated expression in the posterior oral compartment included cadherins *Cdh20* (17FC), and *Cdh18* (4FC), the leucine rich repeat genes *Lrrtm1* (9FC), *Lrrc4c* (9FC), *Lrrtm3* (6FC), and *Lrrc3b* (6FC), the adenylate cyclase *Adcy8* (10FC), *Enpp1* (6FC), *Ncam2* (6FC), the growth factors *Fgf18* (5FC), *Fgf10*, (4FC), *Sema5b* (4FC), *Pdfd* (5FC), *Bmp4* (3FC), as well as Bmp inhibitors *Grem1* (5FC) and *Grem2* (3FC), and transcription factors *Osr1* (4FC), *Osr2* (3FC), *Foxd1* (3FC), *Foxf2* (4FC), *Npas3* (3FC) and many other genes of interest (S5 Table). Genes with elevated expression in the nasal compartment included *Ibsp* (83FC), *Panx3* (40FC), *Dlx3* (40FC), *Hrc* (18FC), *Smpd3* (15FC), *Slc8a3* (11FC), *Sp7* (9FC), *Sox6* (7FC), *Tcf7* (5FC), *Dlx5* (5FC), *Bmp6* (5FC), *Tnn* (4FC), *Pax3* (4FC) and *Gdf10* (4FC).

### Regulatory genes with high expression levels

The dataset can also be screened to find genes with particularly strong expression, even though not necessarily compartment specific. Important functionality is not always related to differential expression. Transcription factor genes with RPKM expression levels over 50 in at least one compartment include *Sirt1*, *Foxf2 Twist1*, *Runx2*, *Nfatc4*, *Myc*, *Tcf3*, *Tcf4*, *Tcf12*, *Tcf7l1*, *Shox2*, *Sox9*, *Sox11*, *Ezh2*, *Tcea1*, *Pax3*, *Lhx6*, *Lhx8*, and *Ski*. Growth factor genes with strong expression include *Nenf*, *Mdk*, *Hdgf*, *Ptn*, *Cxcl12*, *Igf1*, *Igf2*, *Inhba*, *Ogn*, *Bmp4*, *Gmfb* and *Vegfb*.

### Validations

The dataset described in this report provides a global view of the gene expression patterns of the multiple compartments of the developing palate. A comparison of this dataset with previously published expression patterns for specific genes provides a key measure of validation. For example, *Fgf10* has been reported expressed more in the lateral and oral compartments, compared to medial and nasal [17]. The RNA-Seq data shows *Fgf10* up 4.2 fold in the posterior oral versus nasal, up 2.3 fold in anterior oral versus nasal, up 4.9 fold in anterior lateral versus medial, and up 8.6 fold in the posterior lateral versus medial. Similarly, *Gli1* in situ hybridizations show stronger expression in oral/lateral compartments [17], and the RNA-seq data shows *Gli1* up 1.8 fold in posterior lateral versus medial, up 1.75 fold in anterior lateral versus medial, and up 3.2 fold in anterior oral versus nasal compartments. Conversely, *Dlx5* is expressed more strongly in the nasal compartment compared to oral [17]. Consistent with this the RNA-seq data shows a 5.2 fold elevated expression of *Dlx5* in the posterior nasal versus oral compartments, and an even stronger 11 fold higher expression in the anterior nasal versus oral compartments. *In situ* hybridizations show *Ctgf* expression higher in the medial compartment [18], further confirming the RNA-seq data of this report, which shows *Ctgf* four fold higher in the anterior medial versus lateral compartment, with much stronger expression in the anterior versus posterior palate. Previous *in situ* hybridizations show *Osr1* with stronger expression in the lateral compartment [19], again consistent with the RNA-Seq data, which shows 4.7 fold higher expression in the anterior lateral versus medial compartments, and 3 fold elevated expression in the posterior lateral compartment. *Runx2* expression has been shown elevated in the nasal versus oral compartment [20], agreeing with the RNA-seq data of this report, which shows 3.1 fold higher expression in the anterior and 2.3 fold elevation in the posterior nasal compartment. The RNA-Seq data shows *Frzb* elevated expression elevated 2.1 fold in the posterior lateral versus medial compartments, consistent with published expression data [21]. Once again congruent with published data *Sox9* showed 5.2 fold elevated expression in the anterior medial versus lateral compartments [22]. The RNA-seq dataset is further confirmed by the published expression of *Tnn* in the nasal compartment [23], with the RNA-seq data showing 4.4 fold higher expression in the posterior nasal versus oral, and 6.7 fold elevated expression in the anterior nasal versus oral compartments. Other confirmations for previously published anterior/posterior gene expression differences were mentioned earlier. More examples of agreement between this RNA-seq dataset and previously described palate development gene expression patterns can be found at FACEBASE.ORG [15]. In summary, the RNA-seq dataset provided in this report is highly concordant with the extensive published gene expression literature for palate development.

**Fig 7:**
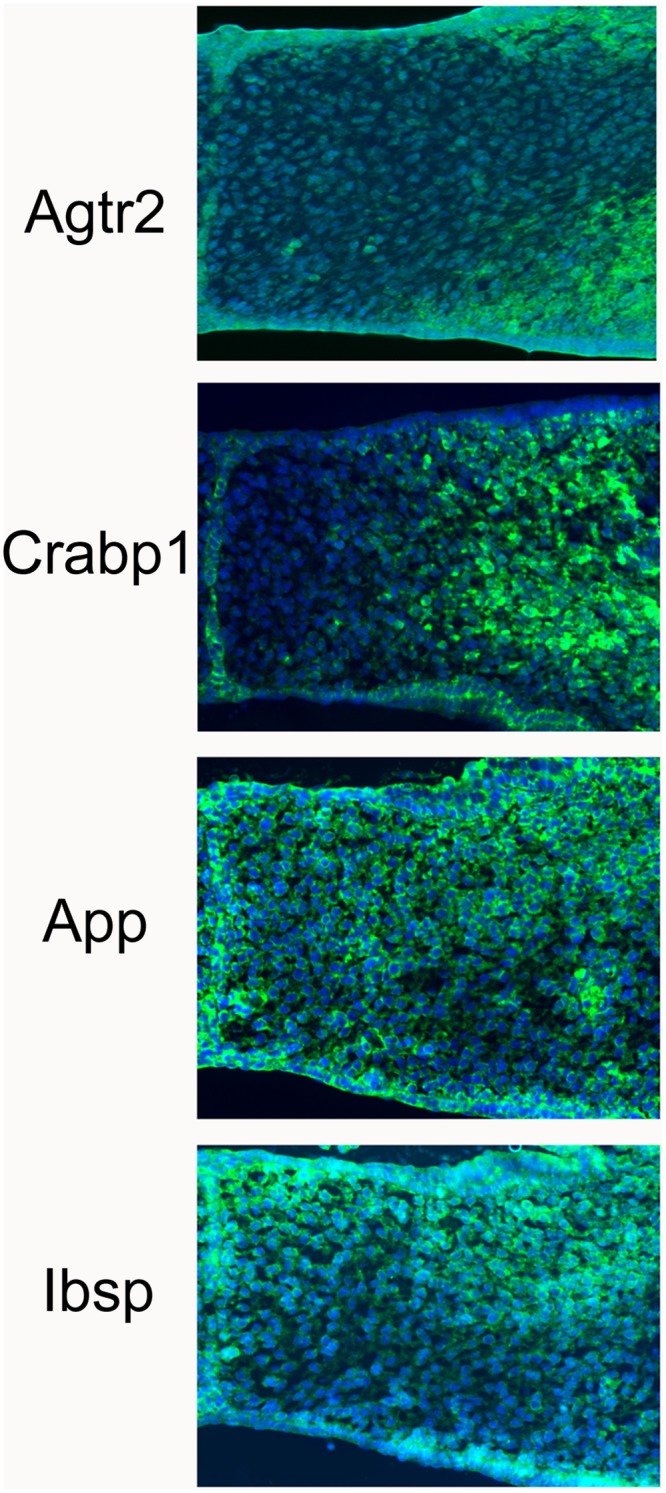
Imunostain validations of RNA-Seq predicted gene expression patterns. The tissues sections shown are of one half of the E14.5 forming palate, with the medial edge epithelium at the left side of the image. For Agtr2 the RNA-Seq strongest expression was in the lateral and oral compartments, consistent with the immunostain results. Crabp1 immunostain showed strongest expression in the lateral compartment, as predicted by RNA-Seq. Both App and Ibsp showed strongest expression in the nasal compartment (above), compared to oral compartment (below), as predicted by RNA-Seq.

We further validated the RNA-seq dataset by carrying out selected immunostains. For Agtr2 we observed elevated expression in the lateral and oral compartments, as predicted by the RNA-seq (Fig 7). Similarly, Crabp1 showed elevated expression in posterior lateral versus medial, and both App1 and Ibsp were more strongly expressed in nasal versus oral compartments, again as predicted by the RNA-seq data.

### Cleft palate associated genes

One potential use of the gene expression data presented in this report is to aid in the screening of DNA sequencing data from patients for causative mutations associated with cleft palate. Genes with appropriate expression during palate formation would represent preferred candidates. To this end it is interesting to examine genes previously associated with cleft palate to determine the types of expression patterns represented. A search of the GATACA website (https://gataca.cchmc.org/gataca/) identifies 91 human genes related to some degree with cleft palate. It should be noted that this is a broadly inclusive list, and not all of the cleft palate gene associations are strong. Of these 91 genes 20 showed no expression in the developing E14.5 murine plate. Of course these genes could still be functionally important and expressed at other critical stages of palate development. Nevertheless, they would represent weaker candidates. Another 14 genes, including *Bmp4*, *Satb2*, *Runx2*, *Msx1*, *Sox9*, *Tfap2a*, *Fras1*, *Alx4*, *Tbx22*, *Sema3e*, *Ptch1*, *Irf6*, *Fgfr2* and *Eya1* showed expression that varied among the E14.5 palate compartments. The predominant expression pattern observed, however, was uniform expression throughout the developing palate. In some cases this expression was at high levels (over 50 RPKM), for *Yhae*, *Sumo1*, *Ubb*, *Twist1*, *Gpc3*, *Flna*, *Col11a1* and *Fgfr1*. For most of the cleft associated genes, however, gene expression was relatively uniform throughout all palate compartments and at moderate levels (5–50 RPKM). The subset of genes with the strongest cleft palate associations also showed multiple expression patterns. For example, *Wnt3* and *Vax1* showed no detectable expression, *Msx1*, *Irf6* and *Bmp4* showed expression that varied among compartments, and *Bmpr1a* showed uniform high expression. Therefore no simple formula for connecting the observed gene expression patterns to cleft palate causation emerged.

**In summary** this report provides an LCM/RNA-Seq based definition of the gene expression programs driving palate development. Lateral and medial, as well as oral and nasal compartments were examined for both the anterior and posterior E14.5 secondary palate. The results provide a global view of the region specific expression of all transcription factors, growth factors and receptors. Paracrine interactions can be inferred from flanking compartment growth factor/receptor expression patterns. The dataset provides a resource for the identification of novel molecular markers of palate development, and for the development of new compartment specific CRE and GFP transgenic tools. This RNA-Seq data contributes to the FACEBASE consortium generation of a molecular anatomy of craniofacial development.

## Materials and Methods

### Animals, Laser capture and RNA purification

Laser capture was carried out with an Arcturus Veritas machine as previously described [16]. Sections were removed from caps and placed in 50 μl of lysis buffer (0.05% SDS, 5.0 mM Tris 7.4). RNA was purified with Qiagen RNEasy Micro Kit, cat. no. 74004, standard protocol, w/DNAse treatment.

This study was carried out in strict accordance with the recommendations of the Guide for the Care and Use of Laboratory Animals of the National Institutes of Health. The protocol (2D12115) was approved by the Institutional Animal Care and Use Committee (IACUC) of Cincinnati Children’s Research Foundation. CD-1 strain mice were used. All efforts were taken to minimize pain experienced by the mice. Animals were sacrificed by carbon dioxide inhalation.

### Immunostains

Immunostains were carried out with TSKit #12, with HRP—Goat Anti-Rabbit IgG and Alexa Fluor 488 Tyramide, T-20922, Life Technologies, stanard protocol for immunohistochemistry. Antibodies used were as follows. Crabp1: CRABP1 (D7F9T) Rabbit mAb #13163, Cell Signalling,1:400 dilution. Ibsp: Rabbit Anti-Bone Sialoprotein II Polyclonal Anitbody (bs-2668R), Bioss, 1:100 dilution. Agtr2: Anti-Angiotensin II Type 2 Receptor antibody (ab19134), Abcam, 1:75 dilution. App: Rabbit Anti-APP/Amyloid Precursor Protein Polyclonal Antibody (bs-0112R), Bioss, 1:50 dilution.

### Data Analysis

Data analysis was carried out with GeneSpring 12.6.1, as previously described [16]. RNA-Seq was single end 50 with an average of 30 million reads per sample. The analysis pipeline included filtering on post alignment read metrics to remove reads with more than one alignment to the genome. Duplicate reads were also removed. Expression level filtering typically required RPKM of at least five in three samples, unless stated otherwise. Statistical tests were carried out with both Audic-Claverie (A-C) pooled analysis, examining for deviation from predicted Poisson distribution, as well as moderated t-test. The lists of differentially expressed genes were typically larger with the A-C test, and for some of the historic validations the predicted differences were only seen with A-C. Except for the AP comparison, however, the supplementary tables were all generated with the moderated t-test. The raw and processed DNA sequencing data is available at GEO (series GSE67525). It is also available through the FACEBASE consortium at FACEBASE.org.

## Supporting Information

S1 Table Gene expression differences between combined anterior and combined posterior compartments. (XLS)

S2 Table Anterior palate, lateral versus medial gene expression differences. (XLS)

S3 Table Posterior palate, lateral versus medial gene expression differences. (XLS)

S4 Table Anterior palate, oral versus nasal gene expression differences. (XLS)

S5 Table Posterior palate, oral versus nasal gene expression differences. (XLS)
